# *MYCN* mediates *TFRC*-dependent ferroptosis and reveals vulnerabilities in neuroblastoma

**DOI:** 10.1038/s41419-021-03790-w

**Published:** 2021-05-19

**Authors:** Yuxiong Lu, Qing Yang, Yubin Su, Yin Ji, Guobang Li, Xianzhi Yang, Liyan Xu, Zhaoliang Lu, Jiajun Dong, Yi Wu, Jin-Xin Bei, Chaoyun Pan, Xiaoqiong Gu, Bo Li

**Affiliations:** 1 Clinical Biological Resource Bank, Guangzhou Institute of Pediatrics, Guangzhou Women and Children’s Hospital, Zhongshan School of Medicine, Sun Yat-sen University, Guangzhou, China; 2 State Key Laboratory of Oncology in South China, Collaborative Innovation Center for Cancer Medicine, Sun Yat-sen University Cancer Center, Guangzhou, China; 3 Department of Biochemistry, Zhongshan School of Medicine, Sun Yat-sen University, Guangzhou, China; 4 Key Laboratory of Functional Protein Research of Guangdong Higher Education Institutes, Department of Biotechnology, College of Life Science and Technology, Jinan University, Guangzhou, China; 5 State Key Laboratory of Translational Medicine and Innovative Drug Development, Simcere Diagnostics Co., Ltd., Nanjing, China; 6 Department of Neurosurgery, Jiangmen Central Hospital, Affiliated Jiangmen Hospital, Sun Yat-sen University, Jiangmen, China; 7 Center for Precision Medicine, Sun Yat-sen University, Guangzhou, China; 8 RNA Biomedical Institute, Sun Yat-sen Memorial Hospital, Sun Yat-sen University, Guangzhou, China

**Keywords:** Cancer metabolism, Cell death

## Abstract

*MYCN* amplification is tightly associated with the poor prognosis of pediatric neuroblastoma (NB). The regulation of NB cell death by *MYCN* represents an important aspect, as it directly contributes to tumor progression and therapeutic resistance. However, the relationship between *MYCN* and cell death remains elusive. Ferroptosis is a newly identified cell death mode featured by lipid peroxide accumulation that can be attenuated by GPX4, yet whether and how *MYCN* regulates ferroptosis are not fully understood. Here, we report that *MYCN*-amplified NB cells are sensitive to GPX4-targeting ferroptosis inducers. Mechanically, *MYCN* expression reprograms the cellular iron metabolism by upregulating the expression of *TFRC*, which encodes transferrin receptor 1 as a key iron transporter on the cell membrane. Further, the increased iron uptake promotes the accumulation of labile iron pool, leading to enhanced lipid peroxide production. Consistently, *TFRC* overexpression in NB cells also induces selective sensitivity to GPX4 inhibition and ferroptosis. Moreover, we found that *MYCN* fails to alter the general lipid metabolism and the amount of cystine imported by System X_c_(−) for glutathione synthesis, both of which contribute to ferroptosis in alternative contexts. In conclusion, NB cells harboring *MYCN* amplification are prone to undergo ferroptosis conferred by *TFRC* upregulation, suggesting that GPX4-targeting ferroptosis inducers or TFRC agonists can be potential strategies in treating *MYCN*-amplified NB.

## Introduction

Neuroblastoma (NB) is the most common extracranial solid tumor in human infants, with 90% of cases diagnosed by the age of 5^1^. Genetically, frequent amplifications of the oncogene *MYCN* are identified in patients with NB, predicting poor prognosis independent of other factors^2^. Although *MYCN* inhibition leads to the suppression of NB in vitro, there are technical challenges in targeting *MYCN* clinically^3,4^. *MYCN* orchestrates multiple molecular pathways for cell growth, survival, metabolism, and death, dictating cancer cell fate and tumor progression^5,6^. In order to explore new treatments against *MYCN*-amplified NB, it is necessary to gain a deeper understanding of the biological functions mediated by *MYCN*.

Among the key cellular processes governed by *MYCN*, cell death is a puzzling phenomenon. On one hand, it is the cause of tumor growth under nutrient-deprived conditions whose adaptation promotes NB progression^7,8^; on other hand, abnormal expression of *MYCN* leads to the direct activation of cell death pathways^9,10^. This contradiction indicates that *MYCN* finely regulates the balance between cell survival and cell death. Recently, researchers discovered a new type of programmed cell death, namely ferroptosis, which is characterized by intracellular iron disorder and accumulation of lipid peroxides in the cell membrane^11^. Cell culture and animal experiments have shown that induction of ferroptosis can eliminate selective tumor cells that are resistant to apoptosis, such as in kidney cancer^12^, breast cancer^13^, lung cancer^14^, etc., which provides a new venue for overcoming drug resistance. System X_c_(−) and GPX4 inhibition are the most common methods to induce ferroptosis. System X_c_(−) imports cystine as a key substrate for synthesizing glutathione (GSH), which is the reducing equivalent used by GPX4 to antagonize ferroptosis^15^. Although *MYCN* has a role in regulating cellular redox balance^16^, the potential effects of *MYCN* on ferroptosis remain unclear.

In this study, we identified a new regulatory mechanism of ferroptosis by *MYCN*. We found that NB cells overexpressing *MYCN* are particularly sensitive to ferroptosis induced by GPX4 inhibition rather than system X_c_(−) blockage. Moreover, *MYCN* amplification fails to cause significant changes in lipid species, suggesting that alternations of iron metabolism are mainly accounting for *MYCN* regulated ferroptosis. Therefore, we analyzed the expression of iron-related genes and revealed that *TFRC*, which encodes transferrin receptor 1 as an essential iron transporter on the cell membrane, plays a key role in the process of *MYCN* regulated ferroptosis. Similar to *MYCN*, *TFRC* significantly increases the intracellular iron load, and *TFRC* upregulation confers cell sensitivity to ferroptosis induced by GPX4 inhibition. Considering that *MYCN* and *TFRC* are both deregulated in a variety of tumor types, our finding points to a tumor vulnerability that can be therapeutically exploited.

## Results

### *MYCN* is a regulator of GPX4-dependent ferroptosis

**Fig. 1:**
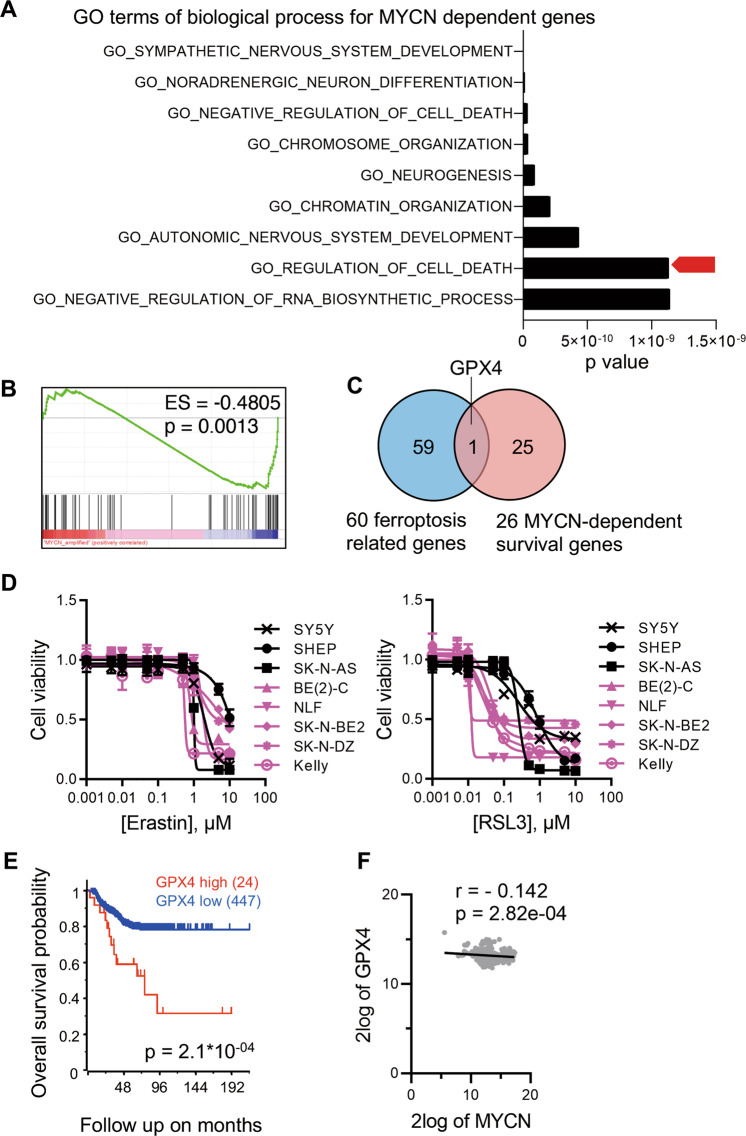
*MYCN* is a regulator of GPX4-dependent ferroptosis. **A** Genes selectively dependent on *MYCN* for NB cell survival are enriched within a variety of GO terms of biological processes, and the top nine processes are shown according to *p* values. **B** GSEA assays of ferroptosis-related genes showing enrichment with *MYCN* amplification based on RNA-seq data of six human NB cell lines. ES, enrichment score. **C** *GPX4* is shared between ferroptosis-related and MYCN-dependent survival genes. **D** Cell viabilities when different concentrations of the GPX4 inhibitor (1S, 3R)-RSL3 (RSL3) and system X_c_(−) inhibitor Erastin were used as ferroptosis inducers in multiple human NB cell lines (*MYCN* amplified SK-N-BE2, BE(2)-C, NLF, SK-N-DZ, and Kelly are represented in pink; and *MYCN* non-amplified SHEP, SK-N-AS, and SY5Y are shown in black; *n* = 4). **E** Kaplan–Meier survival curves for NB patients based on *GPX4* mRNA expression. Gene expression and clinical data were retrieved from GSE45547. **F** Correlation analysis between *GPX4* and *MYCN* expression based on the data retrieved from GSE45547. *p < 0.05, **p < 0.01.

To study *MYCN*-dependent cellular processes in NB, we examined a collection of 147 genes essential for the survival of *MYCN*-amplified NB cells^17^ through pathway analysis using the Molecular Signatures Database v7.2. We discovered that genes regulating cell death are significantly enriched in this collection (Fig. 1A). Furthermore, we performed RNA-seq and gene set enrichment analysis (GSEA) of six human NB cell lines (*MYCN* amplified SK-N-BE2, BE(2)-C, NLF, SK-N-DZ, and *MYCN* non-amplified SHEP, SK-N-AS), and found that *MYCN* expression is closely associated with levels of ferroptosis-related genes as summarized by Liang et al.^18^ (Fig. 1B and Supplementary Table 1). Next, we compared the ferroptosis-related genes with those regulating *MYCN*-dependent cell survival (Supplementary Table 2), and identified *GPX4* as the only overlapping gene (Fig. 1C). GPX4 is a well-established ferroptosis regulator neutralizing the oxidized lipid species^19^. To confirm the above results, multiple NB cell lines were treated respectively with two classic ferroptosis inducer, (1 S, 3 R)-RSL3 (hereafter RSL3 for short), a highly specific GPX4 inhibitor, and Erastin, a cystine-glutamate antiporter (system X_c_(−)) inhibitor. Interestingly, *MYCN* amplification significantly increases the sensitivity of NB cells to RSL3, whereas all NB cells exhibit similar resistance to Erastin (Fig. 1D and Fig. S1A). Consistently, the effects of an alternative system Xc(−) inhibitor sulfasalazine (SAS)^20^ are weakly associated with *MYCN* amplification (Fig. S1B, C). These findings imply that *MYCN* is a key regulator of ferroptosis, and GPX4 might play an essential role in this process. Indeed, high expression of *GPX4* in NB tumor tissues predicts poor prognosis (Fig. 1E). However, RNA-seq data reveal that there is no significant correlation between *GPX4* and *MYCN* expression (Fig. 1F and Fig. S1D). Therefore, *MYCN* regulates GPX4-dependent ferroptosis likely through an unknown mechanism.

### *MYCN* confers cell sensitivity to ferroptosis upon GPX4 inhibition

**Fig. 2:**
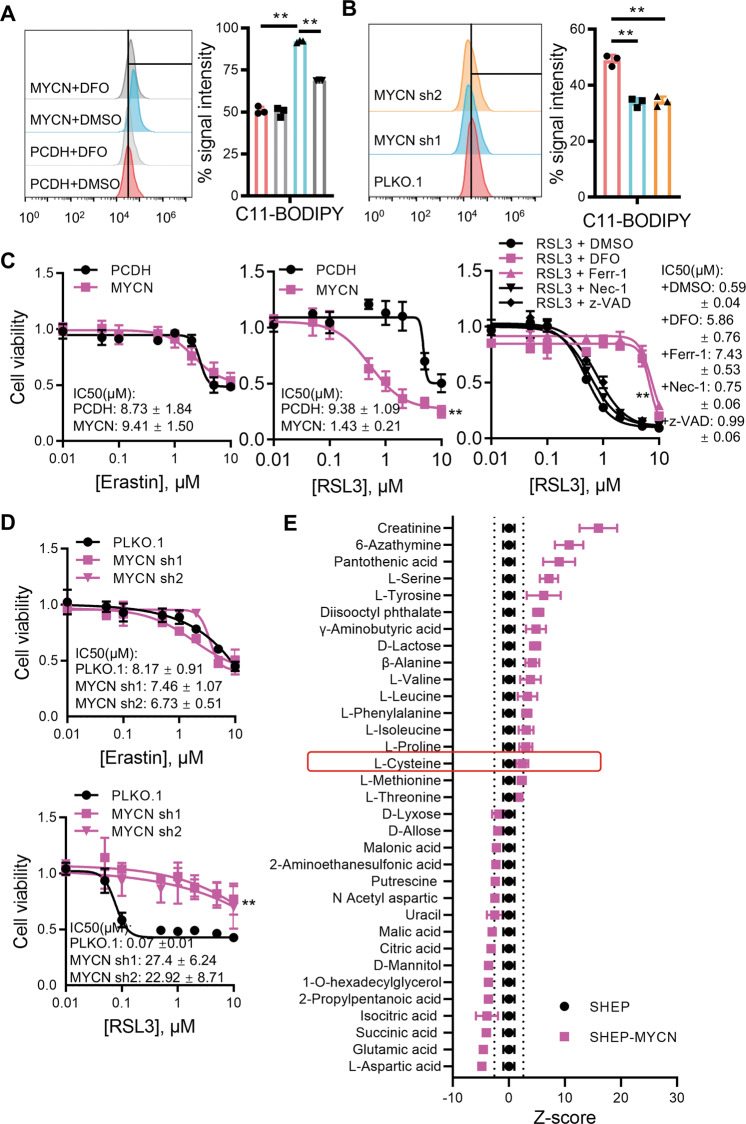
*MYCN* confers cell sensitivity to ferroptosis upon GPX4 inhibition. Lipid peroxides were detected by flow cytometry after incubation with C11-BODIPY in SHEP (**A**) or SK-N-BE2 (**B**) cells with or without *MYCN*. DFO, desferrioxamine, *n* = 3. **C** Cell viabilities of SHEP cells with or without MYCN overexpression incubated with Erastin (left), RSL3 (middle), or RSL3 combined with either ferroptosis inhibitor (10 μM DFO, 1 μM Ferr-1), apoptosis inhibitor (20 μM z-VAD), or necroptosis inhibitor (2 μM Necrostatin-1) (right). **D** Cell viabilities of SK-N-BE2 cells with or without MYCN knockdown incubated with Erastin or RSL3. **E** Metabolic analysis of SHEP cells with or without *MYCN*. Z-score ±2.75 is corresponding to *p* = 0.01 and depicted as a dotted line. Data are presented as mean ± SD of three replicates. Two-tailed unpaired *t*-tests were performed to calculate *p* values. **p < 0.01.

To confirm that *MYCN* is linked to ferroptosis, we ectopically expressed MYCN in SHEP, a *MYCN* non-amplified NB cell line, and found that MYCN expression significantly increases lipid peroxide contents. Conversely, lipid peroxides are reduced when *MYCN* is depleted in SK-N-BE2, a *MYCN* amplified NB cell line (Fig. 2A, B). Next, MYCN overexpression significantly increases the sensitivity of SHEP cells to RSL3, but not Erastin. Moreover, *MYCN* induced sensitivity towards RSL3 can only be rescued by treating cells with ferroptosis inhibitors, but not other cell death inhibitors (Fig. 2C). These results are further supported by the data in *MYCN* amplified SK-N-BE2 cells with shRNA-mediated *MYCN* depletion (Fig. 2D).

The function of GPX4 is to convert toxic lipid peroxides to nontoxic alcohol lipids using the reducing equivalent glutathione. The system X_c_(−) imports cystine, the extracellular form of cysteine, for glutathione synthesis, therefore functioning upstream of GPX4. To test whether the intracellular cysteine level can be regulated by *MYCN*, we use mass spectrometry to identify altered metabolites upon *MYCN* overexpression, and found that *MYCN* does not significantly affect the intracellular cysteine level (Fig. 2E). Meanwhile, our metabolic analysis confirms that *MYCN* amplification elevates serine levels, which is consistent with previous findings such as by Xia et al.^21^. These results demonstrate that cellular cystine import is independent of *MYCN*. Together, we conclude that *MYCN* induces NB cell sensitivity to ferroptosis upon GPX4 inhibition, through a mechanism non-correlated with cysteine metabolism.

### *MYCN* increases the intracellular iron load

**Fig. 3:**
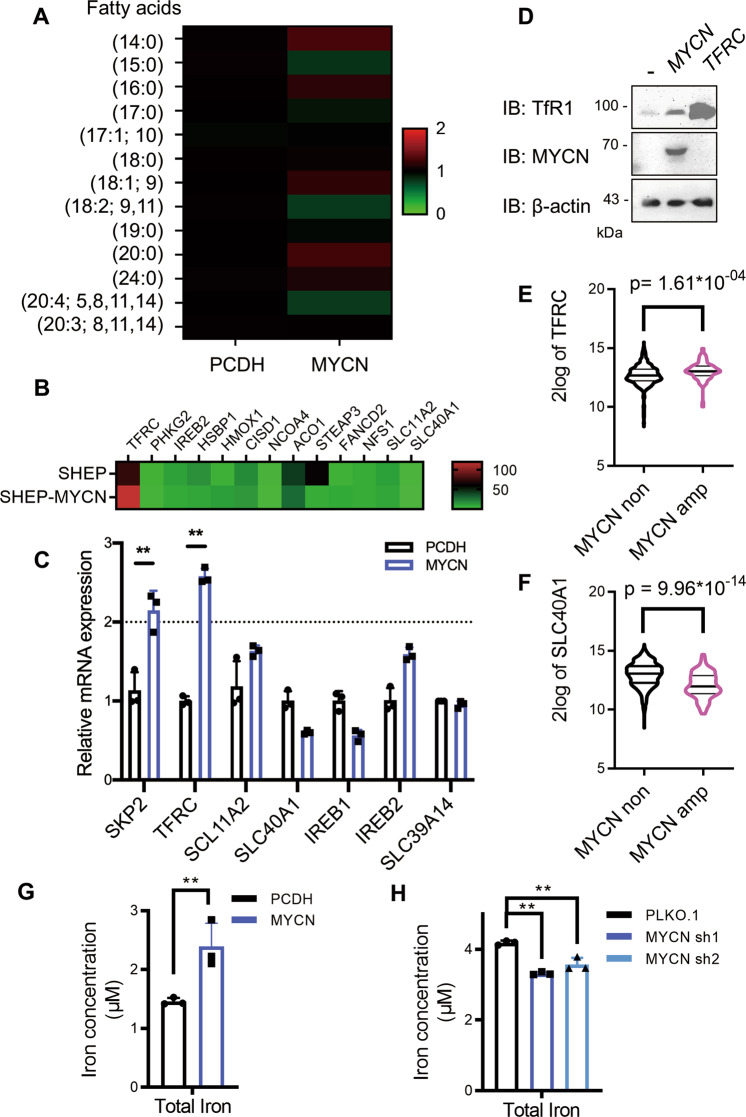
*MYCN* increases the intracellular iron load. **A** Heatmap showing changes in an array of fatty acids in SHEP cells with or without *MYCN*. Data are shown as relative changes in abundance compared to the PCDH vector. **B** Heatmap showing the expression profile of ferroptosis-associated metabolic genes. **C** Expression of *MYCN* induced iron metabolic genes analyzed by RT-qPCR, and *SKP2* is positive control of *MYCN* targeted genes. **D** Western blot analysis of SHEP cells with or without *MYCN* expression using indicated antibodies. **E**, **F** *TFRC* and *SLC40A1* expression in NB tumor tissues with or without *MYCN* amplification. Expression data were retrieved from GSE45547. Cellular iron load is detected with the colorimetric ferrozine-based assay in SHEP **G** and SK-N-BE2 **H** cells with or without MYCN, and values are normalized to protein concentration. Data are presented as mean ± SD of three replicates. Two-tailed unpaired *t*-tests were performed to calculate *p* values. **p < 0.01.

Deregulated iron metabolism and abnormal lipid synthesis are both required to induce ferroptosis^22^. To this end, we examined the fatty acid content upon *MYCN* overexpression by mass spectrometry and found that *MYCN* has no significant influence on cellular fatty acid pools (Fig. 3A). Next, we analyzed the expression of several ferroptosis-related genes as summarized by Hassannia et al.^23^, and identified *TFRC* as the most upregulated one both in RNA-seq data (Fig. 3B) and RT-PCR results (Fig. 3C). *TFRC* encodes transferrin receptor 1 (TfR1), which is the prevalent iron importer on the cell membrane. Consistently, *MYCN* upregulation enhances the protein level of TfR1 in NB cells (Fig. 3D), which parallels with *TFRC* levels in NB tumor tissues (Fig. 3E). Interestingly, we noticed that the expression of *SLC40A1*, which encodes the only known iron exporter ferroportin, is slightly but reproductively reduced upon *MYCN* overexpression (Fig. 3C), and RNA-seq analysis of NB tumor tissues also supports this argument (Fig. 3F). These results suggest that *MYCN* tends to increase the iron load in NB cells. To confirm this, we tested the level of the total iron pool (ferric plus ferrous ion) upon *MYCN* manipulation. As expected, *MYCN* overexpression increases the iron level in SHEP cells, whereas *MYCN* depletion reduces the iron level in SK-N-BE2 cells (Fig. 3G, H). These results indicate that *MYCN* mediates GPX4-dependent ferroptosis likely via regulating iron metabolism.

### *TFRC* photocopies *MYCN* to increase the labile iron pool

**Fig. 4:**
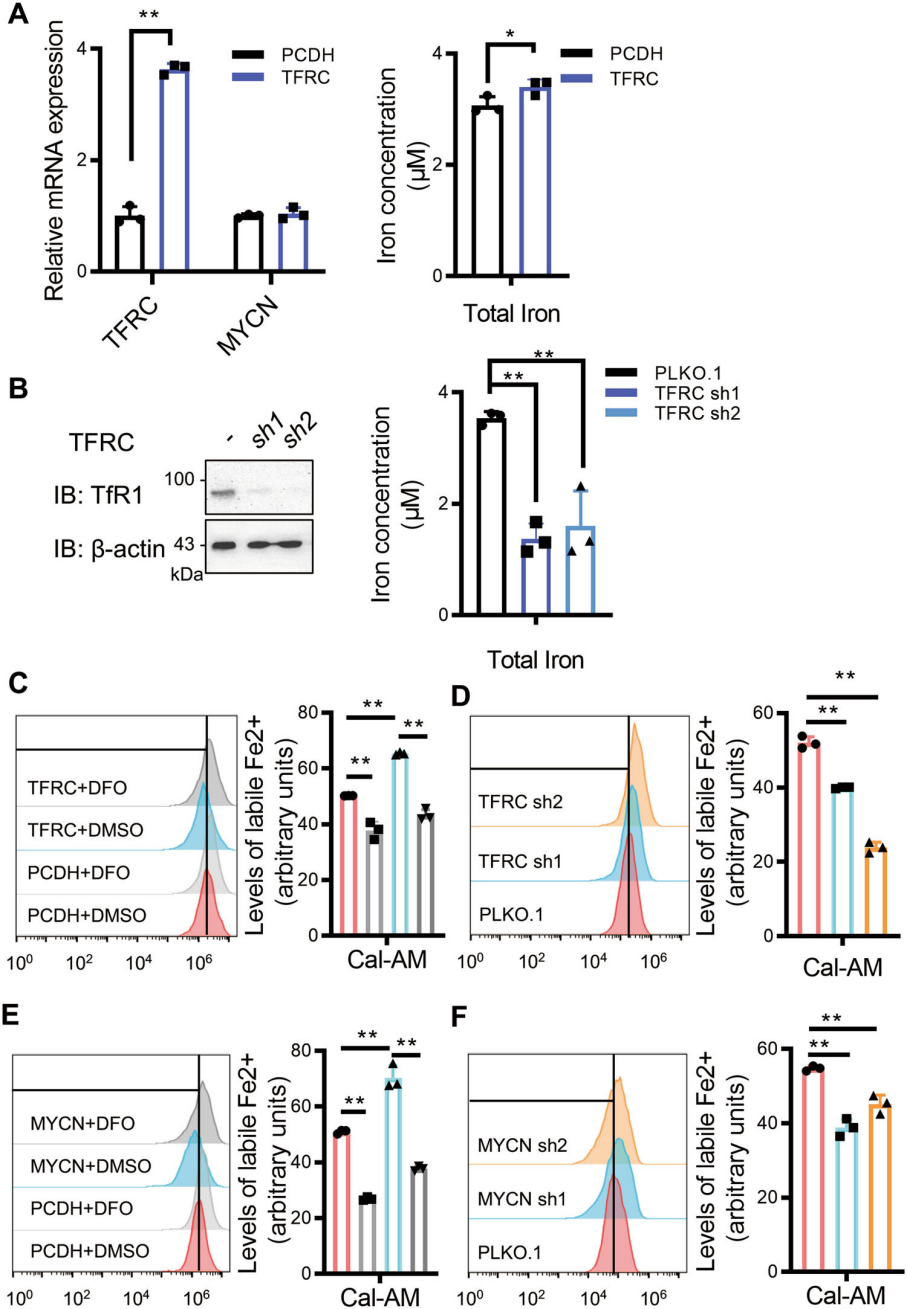
*TFRC* photocopies *MYCN* to increase the labile iron pool. **A** Iron load and RT-qPCR analysis of SHEP cells with or without *TFRC* overexpression. **B** Iron load and western blot analysis of SK-N-BE2 cells with or without *TFRC* depletion. Cellular labile iron pool (LIP) was detected using the probe calcein acetoxymethyl ester (Cal-AM) in SHEP (**C**) and SK-N-BE2 (**D**) cells with or without *TFRC*. LIP was similarly detected in SHEP (**E**) and SK-N-BE2 (**F**) cells with or without *MYCN*. Data are presented as mean ± SD of three replicates. Two-tailed unpaired *t*-tests were performed to calculate *p* values. *p < 0.05, **p < 0.01.

To confirm that *MYCN*-induced *TFRC* expression promotes iron uptake, we overexpressed *TFRC* in SHEP and depleted *TFRC* in SK-N-BE2 cells, respectively. Indeed, *TFRC* expression levels correlate with the cellular iron load (Fig. 4A, B). The Fe^3+^ ion transported by TfR1 undergoes endocytosis, reduction to Fe^2+^, and importation to the cytosol composing the labile iron pool (LIP). Fe^2+^ promotes the formation of lipid peroxides by Fenton reactions. Based on these knowledges, we reasoned that increased cellular iron load would result in LIP upregulation, thereby promoting lipid peroxide formation. To test this, we detected intracellular LIP levels with the calcein acetoxymethyl ester (Cal-AM) probe as previously described^24^. Indeed, the LIP levels are increased upon *TFRC* overexpression and deceased upon *TFRC* knockdown (Fig. 4C, D), which photocopy the effects of *MYCN* (Fig. 4E, F). Therefore, *MYCN* selectively sensitizes NB cells to ferroptosis by increasing the intracellular LIP.

### *TFRC* mediates *MYCN* induced, GPX4-dependent ferroptosis

**Fig. 5:**
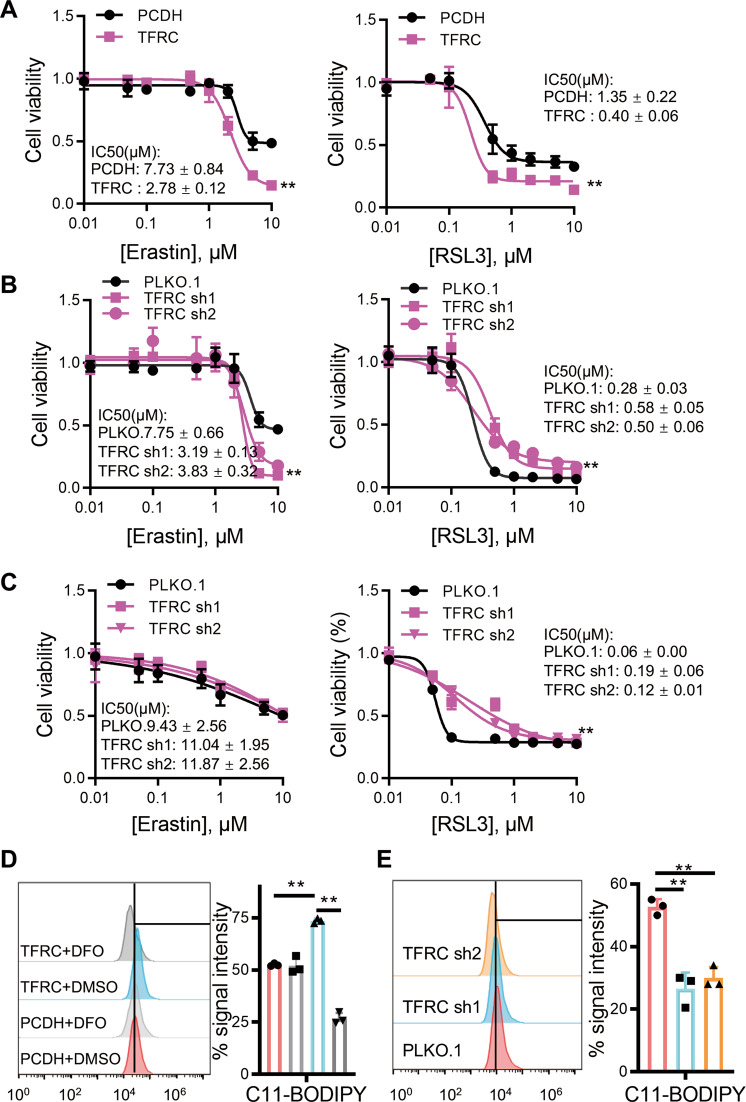
*TFRC* mediates *MYCN*–induced, GPX4-dependent ferroptosis. **A** Cell viability of SHEP cells with or without *TFRC* overexpression incubated with Erastin (left) or RSL3 (right). Cell viabilities of SHEP (**B**) or SK-N-BE2 (**C**) cells with or without *TFRC* depletion incubated with Erastin (left) or RSL3 (right). Amounts of cellular lipid peroxides measured by C11-BODIPY in SHEP (**D**) or SK-N-BE2 (**E**) cells with or without *TFRC*. Data are presented as mean ± SD of three replicates. Two-tailed unpaired *t*-tests were performed to calculate *p v*alues. **p < 0.01.

Our data have shown that *MYCN* upregulates *TFRC* expression, thereby regulating ferroptosis. Next, we explored whether *TFRC* mediates GPX4-dependent ferroptosis, in a similar way as *MYCN*. In *MYCN* non-amplified SHEP cells, we found that *TFRC* overexpression makes cells sensitive to ferroptosis induced by both System X_c_(−) and GPX4 inhibition (Fig. 5A), whereas *TFRC* knockdown only rescues ferroptosis induced by GPX4 inhibition, but not system X_c_(−) inhibition (Fig. 5B). In SK-N-BE2 cells with amplified *MYCN*, we also found that knockdown of *TFRC* rescues the GPX4-dependent but not X_c_^−^-dependent ferroptosis, and the rescue effects are substantially enhanced (Fig. 5C). Moreover, we found that the system Xc(−) inhibitor SAS upregulates *TFRC* expression, and *TFRC*-depleted, *MYCN*-overexpressed NB cells exhibit high resistance to SAS (Fig. S1E–G). The above results suggest that *TFRC* indeed mediates GPX4-dependent ferroptosis, which can be further reinforced by *MYCN* amplification. Consistent with it, the lipid peroxide amount is significantly elevated with ectopic *TFRC* expression, and decreased upon *TFRC* knockdown (Fig. 5D, E).

### *TFRC* is a direct downstream target of *MYCN*

**Fig. 6:**
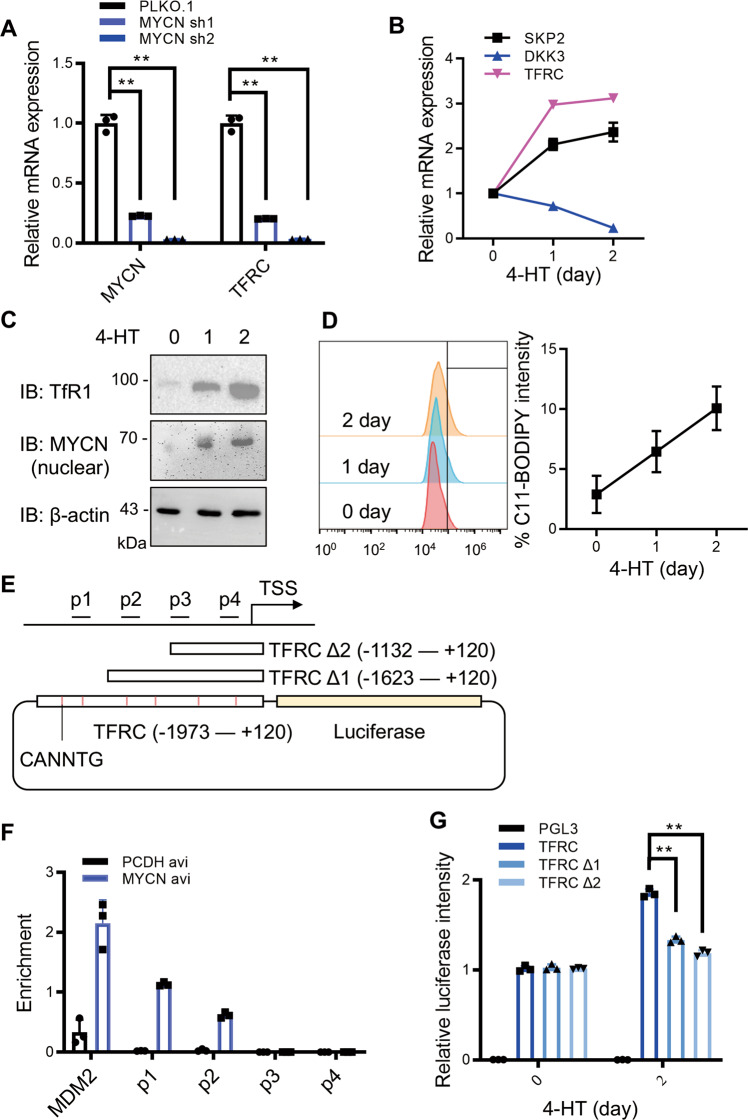
*TFRC* is a direct downstream target of *MYCN*. **A** RT-qPCR analysis of SK-N-BE2 cells with or without *MYCN* depletion. *MYCN* activation induced by 4-hydroxytamoxifen (4-HT) treatment promotes *TFRC* expression at both mRNA **B** and protein **C** levels. *SKP2, DKK3* were used as positive and negative controls, respectively. **D** Amounts of cellular lipid peroxides measured by C11-BODIPY upon *MYCN* activation. **E** Primers designed for ChIP-qPCR analysis, and sequence variants for luciferase assays based on the *TFRC* promoter region. **F** ChIP-qPCR analysis of different binding sites of *MYCN* on the *TFRC* promoter. *MDM2* is a positive control for *MYCN* binding. **G** Dual-luciferase assays based on (**E**). Signal values were normalized to no 4-HT treatment (day 0). Data are presented as mean ± SD of three replicates. Two-tailed unpaired *t*-tests were performed to calculate *p* values. **p < 0.01.

Given that *MYCN* is a well-established transcription factor, we speculated that *MYCN* drives *TFRC* expression through direct transcriptional regulation. Indeed, we confirmed that *TFRC* expression is responsive to either up or down-regulation of *MYCN* (Figs. 3C and 6A). Then, we established an inducible *MYCN*–ER chimeric protein expression system in SHEP cells, in which nuclear translocation and activity of MYCN can be induced by 4-hydroxytamoxifen (4-HT) treatments. Upon 4-HT stimulation, *TFRC* expression is elevated at both mRNA and protein levels (Fig. 6B, C), paralleled with lipid peroxide formation in a time-dependent manner (Fig. 6D). To further dissect exactly how *MYCN* regulates *TFRC* expression, we performed ChIP-qPCR assays and found that *MYCN* mainly binds to the P1 and P2 regions, which are located ~1.5 kb away from the transcription starting site (TSS) within the *TFRC* promoter (Fig. 6E, F). In addition, we cloned the *TFRC* promoter region from −1973 to +120 bp surrounding the TSS as shown in Fig. 6E and generated corresponding truncations. We found that *MYCN*-promoted luciferase signals are strongly attenuated when the region of −1973 to −1623 is eliminated (Fig. 6G), demonstrating that *MYCN* regulates *TFRC* expression by binding to this region. Therefore, we conclude that *MYCN* induces GPX4-dependent ferroptosis by upregulating *TFRC* as a direct downstream target.

## Discussion

The *MYC* family member *MYCN* plays important role in the development of the central nervous system^25,26^, and its abnormal expression correlates with the poor prognosis of NB patients. Meanwhile, elevated activity of *c-MYC*, the predominant *MYC* isoform, also accelerates the progression of low-grade NB^27^, indicating that *MYC* family members share similar functions during NB development. Ferroptosis is a novel type of programmed cell death characterized by iron-dependent, unleashed lipid peroxidation, which has been identified in recent years. Cystine deprivation rendered by system X_c_(−) blockage, or redox imbalance by GPX4 inhibition are two classic approaches to induce ferroptosis^15^. Our study revealed that *MYCN* sensitizes NB cells to ferroptosis, which is largely dependent on GPX4 inhibition rather than cystine deprivation. This is mainly because the abnormal level of *MYCN* stimulates the expression of *TFRC*, increases the intracellular levels of iron load and LIP, ultimately leads to enhanced lipid peroxidation and a tendency towards ferroptosis.

One important function of *MYCN* in NB is upregulating glutamine transporters, such as ASCT2, thereby promoting glutamine addiction^28,29^, a hallmark of tumor metabolic reprogramming. In NB cells, the glutamic acid level is decreased upon *MYCN* amplification (Fig. 2E), confirming that *MYCN* promotes glutamine consumption. It was reported that glutaminolysis and iron are both essential for Erastin-induced ferroptosis in an ischemia/reperfusion model^30^, implying that *MYCN* might induce ferroptosis through system X_c_(−) in that context. However, our results reveal that *MYCN* induces ferroptosis in NB cells independent of system X_c_(−), suggesting that the molecular mechanism underlying *MYCN*-induced ferroptosis is highly context-dependent.

Notably, Floros et al. recently reported that *MYCN*-amplified NB cells are more sensitive to the system X_c_(−) inhibitor sulfasalazine (SAS)^31^. Indeed, *MYCN*-amplified NB cells are slightly sensitive to SAS as reflected by their smaller IC50 values compared to non-amplified cells, although no statistical significance was achieved in our hands (Fig. S1C). This trend is seemingly contradictory to what we observed with Erastin. However, SAS equally inhibits other targets such as NFκB and reduced folate carrier^32,33^ in addition to System X_c_(−), which may help explain this discrepancy. Interestingly, Floros et al. noticed that SAS treatment of *MYCN*-amplified NB in vivo upregulate *TFRC* expression through an uncharacterized mechanism, which can be reproduced by us in vitro (Fig. S1E). Since we demonstrated that enhanced *TFRC* expression is responsible for increased cellular sensitivity to ferroptosis, we reasoned that SAS may exert its effect through a similar mechanism. Indeed, *TFRC*-depleted, *MYCN*-overexpressed NB cells exhibit high resistance to SAS (Fig. S1F, G), demonstrating that SAS sensitizes *MYCN*-amplified NB cells to ferroptosis at least partially through *TFRC* upregulation. This argument can be further supported by the study from Floros et al. For instance, SAS is capable of inducing ~70% cell death in *MYCN*-amplified NB cells. However, knockdown of two System X_c_(−) components SLC7A11 and SLC3A2 induce only ~25% cell death in the same cell line^31^, confirming that SAS harbors considerable off-target effects. In conclusion, the complex effects of SAS treatments leading to ferroptosis cannot be solely linked to the sensitivity of NB cells to System X_c_(−) inhibition.

There are multiple major differences between our studies and the work by Floros et al. First, they were focused on targeting the redox balance of *MYCN*-amplified NB cells to induce ferroptosis, using different small molecule inhibitors such as buthionine-(S,R)-sulfoximine (BSO), sulfasalazine (SAS), and auranofin. All these inhibitors function to antagonize the ROS-scavenging activities in *MYCN*-amplified NB cells. In contrast, our work highlights TFRC as the major downstream target of *MYCN* to confer cell sensitivities to ferroptosis inhibitors. We particularly demonstrated that TFRC depletion greatly suppresses *MYCN*-mediated ferroptosis, as companied by the reduction of the intracellular iron pool. Moreover, *TFRC* overexpression phenocopies *MYCN* amplification to increase the intracellular iron pool and sensitize these tumor cells to ferroptosis. Given that ferroptosis requires both elevated iron levels and dampened ROS-neutralizing activities, these two studies compensate with each other in understanding the vulnerability of *MYCN*-amplified NB cells towards ferroptosis. Second, work by Floros et al. suggested that *MYCN* binds to the *TFRC* promoter and upregulates *TFRC* expression by analyzing relevant data in a public ChIP-seq database. Conversely, we performed ChIP-PCR analyses to identify the *MYCN*-binding region within the *TFRC* promoter, and further confirmed this result by luciferase reporter assays. Third, they found that *MYCN*-amplified NB cells are sensitive to the System X_c_(−) inhibitor SAS, whereas our study suggested that these cells are resistant to the System X_c_(−) inhibitor Erastin.

Iron is an essential element for cell growth and survival, the deregulation of which determines cell fate. In the process of ferroptosis, iron acts as a catalyst for lipid peroxidation through Fenton reactions^23^. TfR1 encoded by *TFRC* is the main iron transporter in cells, and it has been reported that SLC39A14 is an additional iron transporter^34^, although our experiment shows that *MYCN* fails to regulate *SLC39A14* expression (Fig. 3C). In addition, several classical *MYCN* binding motifs (CANNTG) are present in the *TFRC* promoter, and our ChIP-qPCR analysis reveals that *MYCN* precisely binds to a motif-containing region distal to TSS. The specific recognition and regulation of *TFRC* by *MYCN* is consistent with previous findings^35^. Recently, the Stockwell group reported that TfR1 clusters on the cell membrane can be used as a detection marker for ferroptosis^36^. They observed that TfR1 expression is increased during ferroptosis induction, but the mechanism is unknown. Our study supports the MYC signaling as an important candidate. Moreover, it has been reported that TfR1 depletion confers cell resistance to Erastin-induced ferroptosis under abnormal RAS activities^37^. However, we found that *TFRC* knockdown has no significant impact on Erastin-induced ferroptosis in *MYCN*-amplified cells. These results suggest that Erastin-induced ferroptosis harbors a regulatory mechanism independent of *TFRC*, which is likely decided by different oncogenic signals. Although MYC is one of the first identified oncogenes^38,39^, it can function to induce p53-dependent cell death, similar to the case that oncogenic RAS can initiate cell senescence^40,41^. Whether and how different oncogenes cooperate to determine cancer cell fate is pivotal for developing new cancer therapeutics.

## Materials and methods

### Cell culture

Human NB cell lines with *MYCN* amplification (SK-N-BE2, BE(2)-C, NLF, SK-N-DZ, Kelly) and without *MYCN* amplification (SHEP, SK-N-AS, SY5Y) were grown in RPMI 1640 medium supplemented with 10% FBS and 1% penicillin–streptomycin, under 5% CO_2_ at 37 °C. Cell authentication was confirmed by the short tandem repeat analysis, and mycoplasma tests were weekly performed. Cell stocks were created within five passages, and experiments were completed within ten passages.

### Antibodies and reagents

Antibodies and reagents were obtained from commercial sources. Specifically, anti-transferrin receptor, rabbit antibody (ab84036) was from Abcam; N-Myc (D1V2A) rabbit mAb (84406), beta-actin (8H10D10) mouse mAb, HRP-linked mouse IgG (7076 S), and rabbit IgG (7074S) were from Cell Signaling Technology (Massachusetts, USA). Erastin (HY-15763), (1 S,3 R)-RSL3 (HY-100218A), deferoxamine mesylate (DFO), Z-VAD(OMe)-FMK(HY-16658), Necrostatin-1 (HY-15760) were from MedChemExpress (New Jersey, USA); (Z)-4-hydroxytamoxifen (T4420) and sulfasalazine (SAS, T0907) from Target Molecule Corp. (Massachusetts, USA); BODIPY™ 581/591 C11 (D3861), Cal-AM (C3099), Ferrozine (160601), and Neocuproine (N1501) from Invitrogen, Thermofisher (California, USA). Polyethylenimine (PEI) Linear, MW 25000 (24769-2) was from Polysciences Inc (Pennsylvania, USA). CCK-8 reagent is from Beyotime Inst Biotech (C0040, Shanghai, China).

### Constructs

*MYCN* and *TFRC* expression plasmids were constructed by cloning the open reading frame of the corresponding cDNA into the multiple cloning sites of the PCDH vector. shRNA sequences were cloned into PLKO.1 vector according to Addgene’s protocol. For the luciferase assay, promoter sequences were cloned into pGL3 Luciferase Reporter Vectors (E1741, Promega, Wisconsin, USA).

### RNA-seq analysis

Total RNA was extracted from each sample using the standard TRIzol protocol. The Agilent 2100 Bioanalyzer was employed to verify RNA quality. cDNA libraries were generated and processed for sequencing following the Illumina TrueSeq version 2 library preparation kit’s protocol. The cDNA libraries were then sequenced by Illumina HiSeq 2500 with paired-end 2 × 75 bp reads using the HiSeq Control Software (version 2.0.10). The quality of raw reads was evaluated by FastQC, and reads alignment was performed using STAR (version 2.3.0). We used SAMTools to sort the aligned reads, and performed gene-level read quantifications with HTSeq (version 0.10.0).

### Bioinformatic analyses

Gene expression correlation and survival analyses for NB patients (retrieved from GSE45547^42^ and GSE49710^43^) were conducted using the R2 Platform (https://hgserver1.amc.nl/cgi-bin/r2/main.cgi), and the resulting data and p values were calculated according to online instructions. Gene Ontology (GO) analysis of MYCN-dependent genes^17^ was performed using the Molecular Signatures Database v7.2 online tool (http://www.gsea-msigdb.org/gsea/msigdb/annotate.jsp). For GSEA analysis, the expression data of six NB cells by RNA-seq were used, and cell lines were divided into two groups based on the conventional *MYCN* status (*MYCN* amplified: SK-N-BE2, BE(2)-C, NLF, SK-N-DZ v.s. *MYCN* non-amplified: SHEP, SK-N-AS). Lists and expression data of ferroptosis-related and *MYCN*-dependent survival genes are provided in Supplementary Tables 1 and 2.

### Viral production and transduction

HEK-293T cells were seeded on a 10-cm culture dish with 50% confluence. After attachment, cells were co-transfected with the lentiviral expression plasmid (10 μg), viral packaging plasmid (psPAX2, Addgene 12260, 5 μg), and envelope plasmid (pMD2.G, Addgene 12259, 2 μg) using PEI following the manufacturer’s protocols. Viral supernatants were collected and filtered after transfection. For viral transduction, target cells were seeded and incubated with a medium supplemented with viral particles and 8 mg/mL polybrene for 6–8 h. Virus-infected cells were selected with corresponding antibiotics.

### Cell viability assay

Cell viability was detected with CCK-8 according to the manufacturer’s protocol. Briefly, 5 × 10^3^ cells were seeded in 96-well plates per well and treated with different compounds. Twenty-four hours later, cells are incubated with CCK-8 for 2 h at 37 °C. Viability values were obtained by a plate reader at 450 nm.

### Total iron analysis

Total iron concentrations of cells were measured by a colorimetric ferrozine-based assay as previously reported^44^. Briefly, 5 × 10^5^ cells were seeded in 6 cm dishes for 24 h, collected with 50 mM NaOH, and incubate at room temperature (RT) for 2 h. Iron ion was extracted with iron-releasing buffer (1.4 M HCl with 4.5% KMnO_4_, 1:1) at 60 °C for 2 h, followed by incubation with 30 μL detecting buffer (6.5 mM Ferrozine, 6.5 mM Neocuproine, 2.5 M ammonium acetate, 1 M ascorbic acid) at RT for 30 min. Absorbance was measured with a 96-well plates reader at 550 nm. The iron contents were calculated based on FeCl_3_ standard curves and normalized to protein concentration.

### Labile iron pool (LIP) analysis

Calcein acetoxymethyl ester (Cal-AM) is a low-toxic and non-fluorescence dye that can easily pass through the cell membrane. Cal-AM emits fluorescence after being digested by esterase in living cells and quenched in the presence of Fe^2+^, which can be used as a LIP probe as previously described^24^. In this study, 2 × 10^5^ cells were seeded on six-well plates for 24 h, after washing with PBS three times, and incubated with 20 nM Cal-AM in HBSS at 37 °C for 30 min. Then, Cells were trypsinized, suspended, and analyzed using a BD Accuri C6 flow cytometer with a 488 nm laser on an FL1 detector.

### Measurement of lipid peroxides

Cells were incubated with 200 nM C11-BODIPY in HBSS at 37 °C for 30 min, and analyzed using a BD Accuri C6 flow cytometer with a 488 nm laser on an FL1 detector.

### ChIP-qPCR

Assays were performed by following a modified Abcam’s Chromatin immunoprecipitation protocol. Briefly, cells overexpressing biotinylated-tagged proteins (avi tag) were fixed in 1% formaldehyde at RT for 10 min and stopped by the addition of 0.125 M glycine. Cells were then harvested in ChIP Lysis Buffer (50 mM HEPES-KOH pH = 7.5, 140 mM NaCl, 1 mM EDTA, 1% Triton X-100, 0.1% sodium deoxycholate, 0.1% SDS, 1X protease inhibitor cocktail). Lysates were sonicated and DNA sheared to an average length of 200–500 bp. Genomic DNA (Input) was prepared by treating aliquots of chromatin with RNase A, Proteinase K, and 65 °C heat for de-crosslinking, followed by ethanol precipitation. The resuspended pellet was incubated with magnetic streptavidin beads (Z5482, Promega, Wisconsin, USA) for 4 h rocking at 4 °C, washed four times, and eluted from beads with SDS elution buffer (1% SDS, 10 mM EDTA, 50 mM Tris-HCl pH = 8). Crosslinks were reversed by high salt incubation for 4 h at 65 °C. ChIP DNA was purified by phenol–chloroform extraction, followed by quantitative real-time PCR analysis. Primers used for ChIP-qPCR:

TFRC p1: fw: TGAGGTCAGGAATTCGAGACAAG; rev: GAGGAGTGACGGGATTTTATC;

TFRC p2: fw: GCAGCAATGCTCCTGCTCA; rev: GCGACAGAGCAAGATTCCATC;

TFRC p3: fw: GGGAAGAGTAAAAGCCCAAGG; rev: GAACAGCCCTTTAAGAAGCAAATC;

TFRC p4: fw: GGCTGCAAAATACATCTTCACAAG; rev: GCCTTGAAATGTACGTGCAGG;

MDM2 promoter fw: AGCCTTTGTGCGGTTCGTG; rev: CCCCCGTGACCTTTACCCTG.

### Quantitative real-time PCR analysis

Total RNA was extracted from cells using the RNA Midiprep kit (AP-MN-MS- RNA-250, Axygen, California, USA) according to the manufacturer’s protocol. Then, cDNAs were generated using a Reverse Transcription kit (RR036A, Takara, Beijing, China). Quantitative real-time PCR was performed using the TB Green Premix Ex Taq qPCR kit (AQ101, Transgen Biotech, Beijing, China). Primers used for RT-qPCR:

18S: fw: CTACCACATCCAAGGAAGGCAG; rev: TTTTTCGTCACTACCTCCCCG;

MYCN:fw: CCACAAGGCCCTCAGTACC; rev: TCTTCCTCTTCATCATCTTCATCA;

GPX4:fw: TGGGAAATGCCATCAAGTG; rev: GGGGCAGGTCCTTCTCTATC;

SLC11A2: fw: CTGCACCATGAGGAAGAAGC; rev: TGGATACCTGAGTGGCTGAGT;

SLC40A1: fw: CCCCAGCTCTAGCTGTGAAA; rev: CAGGGGTTTTGGCTCAGTAT;

IREB1: fw: CCTCAGCCCCTGTCAAAA; rev: GATTACTGATGGCCACGTGTT;

IREB2:fw: AGAAATATGGTTCAGGAAACTCCA; rev: GCCAAAACAGCTTTCACACC;

SLC39A14: fw: AAGGCCCTACTCAACCACCT; rev: CGACTGCTCGCTGAAATTGTG

TFRC: fw: ACCTGTCCAGACAATCTCCAG; rev: TGTTTTCCAGTCAGAGGGACA;

DKK3: fw: GAAGGAGCCACGAGTGCAT; rev: CCTCATGCTGTCAAGCCAGA;

SKP2: fw: GACGCTATGCACAGGAAGCA; rev: CCTTTAGCAGCTCAGGGAGG.

### Luciferase reporter assay

Dual-Luciferase Reporter Assay System (E1910, Promega, Wisconsin, USA) was used. Briefly, pGL3 vectors carrying *TFRC* promoter sequences were transfected into SHEP cells. Cells were then transferred to six-well plates with 2 × 10^5^ per well. MYCN activity was induced with 40 nM 4-HT treatments. Luciferase signals were measured 48 h later. Firefly luciferase signals were divided by Renilla luciferase signals, and rescaled to set the control signals equal to 1.

Primers for cloning the *TFRC* promoter: fw: CCTGCAAATACCAGCATTGTTTG; TFRCΔ1: fw GAAGGAACACCACAGGGGAGCA; TFRCΔ2 fw: GGCGGTGAGAATCCCAAGTACT; The reverse primer for all above: GACACGAGGGTCGGTGTAGTTC.

### Western blot

Briefly, cells were lysed with RIPA buffer (20 mM Tris-HCl pH7.4, 150 mM NaCl, 1 mM EDTA, 0.5% sodium deoxycholate, 0.1% SDS, 1% Triton-X) supplemented with the protease inhibitor cocktail. Lysates were resolved on SDS-PAGE followed by immunoblotting. Primary antibodies were used following instructions in the antibody dilution buffer (1% BSA, 0.05% sodium azide in 0.05 M TBS, pH = 7.6). ChemiDoc^TM^ Touch Imaging System (Bio-Rad, California, USA) was used to develop the blot on the 0.2 μm diameter PVDF membrane.

### Metabolic analysis

For GC-MS assays, cells were washed with pre-cold physiological saline buffer and harvested in HPLC grade methanol containing internal standards (Ribitol). After sonication and 20,000 g centrifugation at 4 °C, the supernatants were transferred to new 1.5 mL tubes. The supernatants were then transferred into the rotary evaporator and evaporate completely. After treatment with derivatization reagent, samples were analyzed with GC-MS according to the manufacturer protocol. For LC-MS assays, cells were washed with pre-cold physiological saline buffer and harvested in HPLC grade methanol. Then, lipids were extracted with chloroform after 12,000 g centrifugation at 4 °C. The lower phase was evaporated with nitrogen and esterified in 5% sulfuric acid in methanol. The extracts were analyzed by capillary electrophoresis time-of-flight mass spectrometry. Peak areas were normalized to standards and cell numbers.

### Statistical analysis

Data were presented as mean ± SD. Statistical significance was analyzed using a two-tailed unpaired *t*-test and assessed by *p* values as reported in figure legends. Kaplan–Meier srvival analyses employed the log-rank test.

## Supplementary information

Supplementary Figure Legends

Figure S1

Supplementary Table 1

Supplementary Table 2

## Data Availability

Our raw RNA seq deposited in NCBI SRA database, and the accession ID is PRJNA720775.
