# Supplementary material for: MYCN mediates TFRC-dependent ferroptosis and reveals vulnerabilities in neuroblastoma
Source: Cell Death Dis. 2021 May 19;12(6):511. doi: 10.1038/s41419-021-03790-w (PMC8134466; doi:10.1038/s41419-021-03790-w)
Supplement: Supplementary file 1 — Supplementary Figure Legends [file 41419_2021_3790_MOESM1_ESM.docx]

**Supplementary figure and table legends**

**Figure S1: SAS induced ferroptosis is mediated by TFRC.** (**A**) IC50 values of Erastin and RSL3 treated NB cells. (**B, C**) Cell viabilities and IC50 values of indicated NB cells incubated with an alternative system Xc(-) inhibitor sulfasalazine (SAS). (**D**) Correlation analysis between *GPX4* and *MYCN* expression based on the RNA-seq data of 6 NB cell lines. (**E**)TFRC expression in *MYCN* non-amplified SHEP cells treated with 500 μM SAS. (**F, G**) IC50 values of MYCN-overexpressed SHEP cells treated with SAS, in the presence or absence of TFRC shRNAs. Efficacies of TFRC shRNAs were confirmed by RT-PCR. Data are presented as mean ± SD of three replicates. Two-tailed unpaired t tests were performed to calculate p values. ** p < 0.01.

**Supplementary Table 1: RNA-seq FPKM values of 60 ferroptosis related genes in 6 neuroblastoma cell lines.**

**Supplementary Table 2: RNA-seq FPKM values of 26 MYCN-dependent survival genes in 6 neuroblastoma cell lines**
