# Supplementary figures and images for: MYCN mediates TFRC-dependent ferroptosis and reveals vulnerabilities in neuroblastoma
Source: Cell Death Dis. 2021 May 19;12(6):511. doi: 10.1038/s41419-021-03790-w (PMC8134466; doi:10.1038/s41419-021-03790-w)

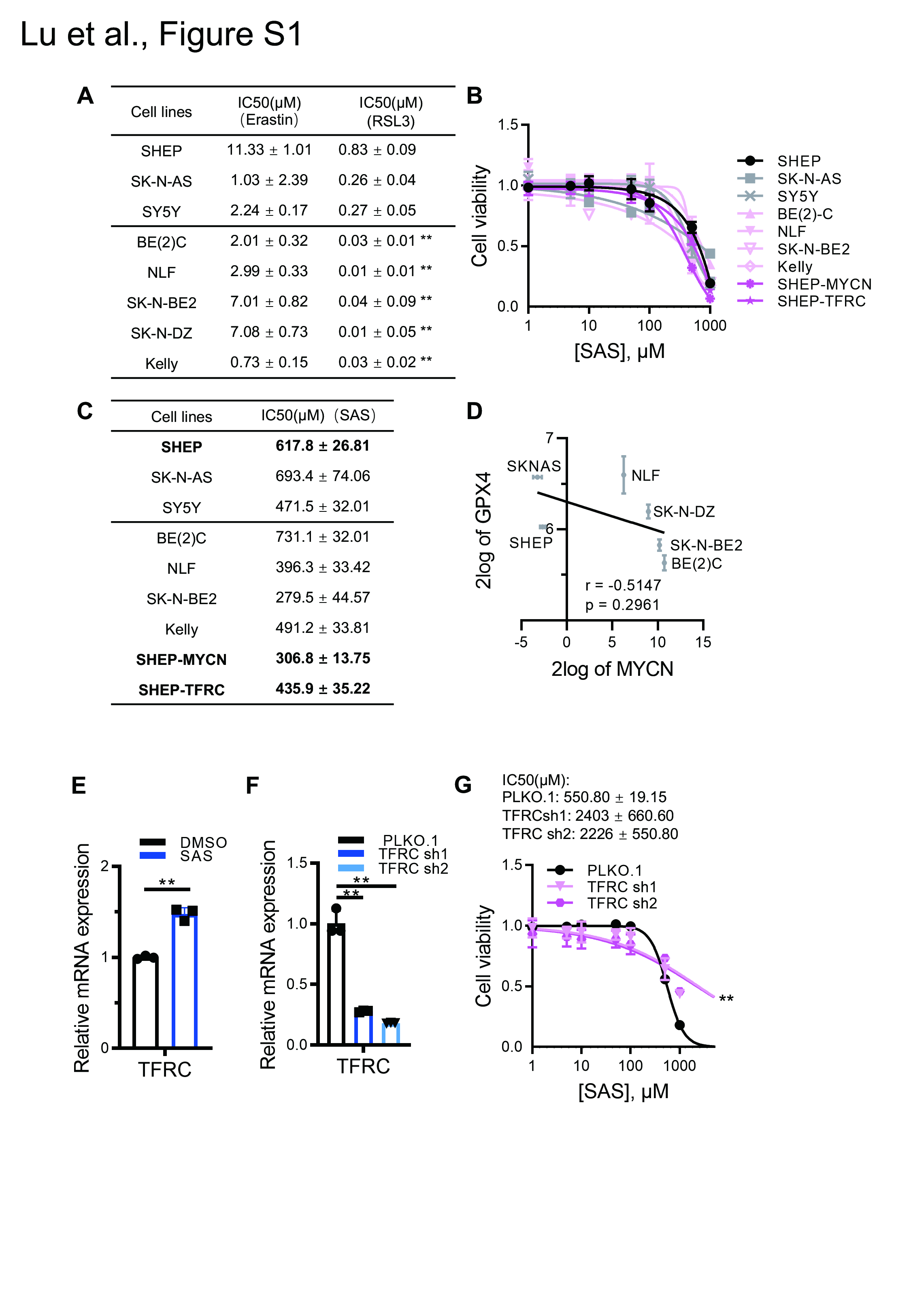

Supplement: Supplementary file 2 — Figure S1 [file 41419_2021_3790_MOESM2_ESM.tif]
